# Tumor Mutation Load: A Novel Independent Prognostic Factor in Stage IIIA-N2 Non-Small-Cell Lung Cancer

**DOI:** 10.1155/2019/3837687

**Published:** 2019-04-30

**Authors:** Jingjing Kang, Yang Luo, Di Wang, Yu Men, Jianyang Wang, Yi-Qun Che, Zhouguang Hui

**Affiliations:** ^1^Department of Radiation Oncology, National Cancer Center/National Clinical Research Center for Cancer/Cancer Hospital, Chinese Academy of Medical Sciences and Peking Union Medical College, Beijing 100021, China; ^2^Department of Medical Oncology, National Cancer Center/National Clinical Research Center for Cancer/Cancer Hospital, Chinese Academy of Medical Sciences and Peking Union Medical College, Beijing 100021, China; ^3^Department of Clinical Laboratory, National Cancer Center/National Clinical Research Center for Cancer/Cancer Hospital, Chinese Academy of Medical Sciences and Peking Union Medical College, Beijing 100021, China; ^4^Department of VIP Medical Services & Department of Radiation Oncology, National Cancer Center/National Clinical Research Center for Cancer/Cancer Hospital, Chinese Academy of Medical Sciences and Peking Union Medical College, Beijing 100021, China

## Abstract

This study is aimed at investigating the prognostic biomarkers of patients with stage IIIA-N2 non-small-cell lung cancer (NSCLC) and at analyzing the correlation between tumor mutation load (the frequency and number of tumor mutations) and prognosis. Clinical data of 35 patients with stage IIIA-N2 NSCLC were collected from Cancer Hospital, Chinese Academy of Medical Sciences. Whole blood samples from the peripheral vein were taken at different treatment periods, and the mutations of cell-free DNA (cfDNA) were detected. Multivariate analysis showed that smoking (*P* = 0.0308), mutation number > 2 (*P* = 0.0283), and max mutation frequency > 0.025 (*P* = 0.0450) were associated with improved progression-free survival (PFS). The overall survival (OS) of well-differentiated NSCLC patients was better than that of poorly differentiated ones (*P* = 0.0006). The rates of PFS, disease-free survival, local-regional recurrence-free survival, and local-regional progression-free survival were significantly higher in the group with a mutation number > 2 than in the group with a mutation number ≤ 2. The mutation number of the preoperation group was significantly higher than that of the postradiochemotherapy group (5 vs. 2.5, *P* = 0.023), and the max mutation frequency change was approximately significant in the postradiochemotherapy group compared with the postoperation group (2.6% vs. 1.85%, *P* = 0.067). The max mutation frequency is positively correlated with vascular invasion (21.13% vs. 3.62%, *P* = 0.04). Furthermore, Met, ALK, APC, PTEN, ERBB4, NF1, and other genes, involving multiple tumor suppressor genes and lung cancer-driven genes, did not mutate in recurrence-free patients when compared with recurrent patients. In conclusion, differentiation, smoking, mutation frequency > 0.025, and mutation number > 2 are prognostic factors. The frequency and number of gene mutations in cfDNA are expected to be prognostic predictors of NSCLC.

## 1. Introduction

According to 2009 international staging of non-small-cell lung cancer (NSCLC), pathological stage IIIA-N2 includes T_1-3_N_2_M_0_ diseases, diagnosed as primary NSCLC with subcarinal lymph node and/or ipsilateral mediastinal lymph node metastasis [1]. The proportion of patients with stage IIIA-N2 NSCLC is about 15% of newly diagnosed cases. For this part of patients, there is still much controversy in clinical treatment strategies. Whether in operation, radiotherapy, and chemotherapy or in different modes of combination therapy, there is lack of an effective standardized therapy [2, 3]. Surgical treatment is used commonly, but the prognosis is poor and the 5-year survival rate was 10%-30% [4]. The stage IIIA-N2 NSCLC is heterogeneous. Different clinical and pathological factors may determine different prognoses and different treatment strategies. Therefore, finding new molecular markers to predict prognosis is crucial to improve the survival of stage IIIA-N2 NSCLC patients.

With the in-depth study of tumor cell biology and molecular biology in recent years in combination with the rapid development of detection techniques, circulating tumor markers are gradually being used to monitor the response during the treatment of tumors due to the convenience and noninvasiveness of detection, which are gradually replacing the histological tumor markers. Cell-free circulating DNA (cfDNA) refers to the DNA fragment released by apoptotic or necrotic cells into blood vessels, mainly in the extracellular plasma, in a free state. The origin of tumor cells in patients with plasma cfDNA is not conclusive, and there are generally two modes: (1) tumor tissue necrosis or apoptosis and DNA release into the blood circulation and (2) active proliferation of tumor cells that were separated into the blood and then dissolved and sustained release of DNA. Several recent studies have shown that the total amount, integrity index, and type and number of tumor-specific mutations of cfDNA can reflect the characteristics of the existence of tumor changes, providing a good target for tumor recurrence and metastasis, efficacy monitoring, and prognosis evaluation [5, 6]. Recent studies have shown that high mutational load is significantly correlated with improved survival in patients with NSCLC [7–9]. However, all these studies were conducted in patients with advance disease and defined high mutational load at various thresholds. Therefore, our study was aimed at assessing the prognostic value of mutational load in patients with stage IIIA-N2 NSCLC and exploring the threshold for prognosis evaluation.

In this project, we retrospectively analyzed the clinical data of 35 patients with stage IIIA-N2 NSCLC and detected cfDNA from peripheral blood collected at different treatment time points. We have thoroughly explored the prognostic factors in patients, which is intended to provide evidence for the development of such strategies and prognosis assessment.

## 2. Methods

### 2.1. Patients and Samples

Patients were recruited at the National Cancer Center/Cancer Hospital, Chinese Academy of Medical Sciences (Beijing, China), between August 2012 and January 2013. Whole blood samples from the peripheral vein were collected from 35 patients with NSCLC. Blood was drawn in K2EDTA tubes (Becton Dickinson) and centrifuged at 1800 × g for 10 min to isolate plasma, which was aliquoted into 1.5-2 mL tubes. And then plasma was further centrifuged at 15,000 × g for 10 min at room temperature to remove residual cells from plasma. All blood samples were stored at -80°C. The whole group of patients did not receive surgery, chemotherapy, or targeting treatment. Written informed consent was provided by all patients prior to enrollment in the study.

### 2.2. DNA Extraction

Cell-free DNA (cfDNA) were extracted from 1~2 mL plasma samples with the QIAamp Circulating Nucleic Acid Kit (Qiagen, Hilden, Germany) according to the manufacturer's instructions. DNA concentration was measured using a Qubit fluorometer (Invitrogen, Carlsbad, CA, USA) and the Qubit dsDNA HS (High Sensitivity) Assay Kit.

### 2.3. Tumor Mutation Load

Mutation frequency refers to a sample of mutation sites, aligned to the location of the sequencing reads divided into two categories: one that supports mutation at the site reads and one that does not support the site mutation reads; the former accounting for the ratio is the mutation frequency. The mutation number refers to the number of mutations detected in a sample. These mutations may occur in the same gene, but the mutations in the same gene are still calculated in multiple ways.

### 2.4. Target Capture and Next-Generation Sequencing

The cfDNA libraries were constructed by using the KAPA Library Preparation Kit (Kapa Biosystems, Wilmington, MA, USA) according to the manufacturer's protocol. Libraries were hybridized to custom-designed biotinylated oligonucleotide probes (Roche NimbleGen, Madison, WI, USA) covering ~1 Mbp of genomic regions in the genes and exons most frequently mutated in solid tumors. DNA sequencing was performed using the HiSeq 3000 Sequencing System (Illumina, San Diego, CA) with 2 × 151 bp paired-end reads.

### 2.5. Sequencing Data Analysis

From raw data, terminal adaptor sequences and low-quality reads were removed. BWA (version 0.7.12-r1039) was employed to align the clean reads to the reference human genome (hg19). Picard (version 1.98) was used to mark PCR duplicates. Realignment and recalibration were performed using GATK (version 3.4-46-gbc02625). Single-nucleotide variants were called using MuTect (version 1.1.4) and NChot and in-house-developed software was used to review hotspot variants. Small insertions and deletions were called by GATK to identify somatic driver mutations with normal sample as the control.

### 2.6. Statistical Analysis

SPSS 22.0 software was used for statistical analysis. The count data were compared using the Fisher exact test, the measurement data were compared using the *t*-test, survival analysis was performed using the log-rank test, and multivariate analysis was performed using the Cox proportional hazards model. Only variables with significant *P* values from univariate analyses were entered into the multivariate analysis. *P* < 0.05 was considered statistically significant.

## 3. Results

### 3.1. Smoking, Mutation Numbers, and Mutation Frequency Affect PFS of Stage IIIA-N2 NSCLC Patients

**Figure 1:**
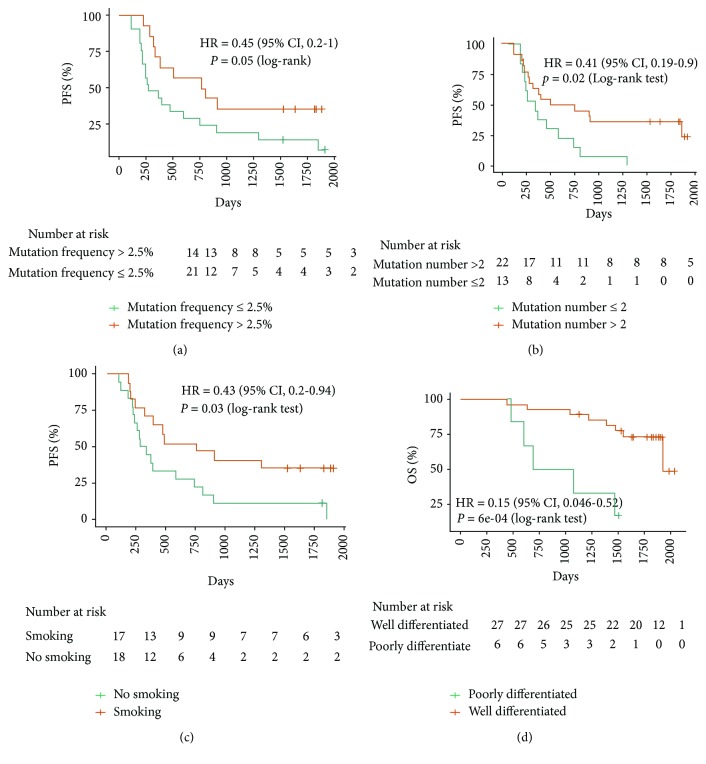
The Kaplan–Meier curves according to risk factors in stage IIIA-N2 NSCLC patients: (a) mutation frequency; (b) mutation number; (c) smoking; (d) tumor differentiation.

**Figure 2:**
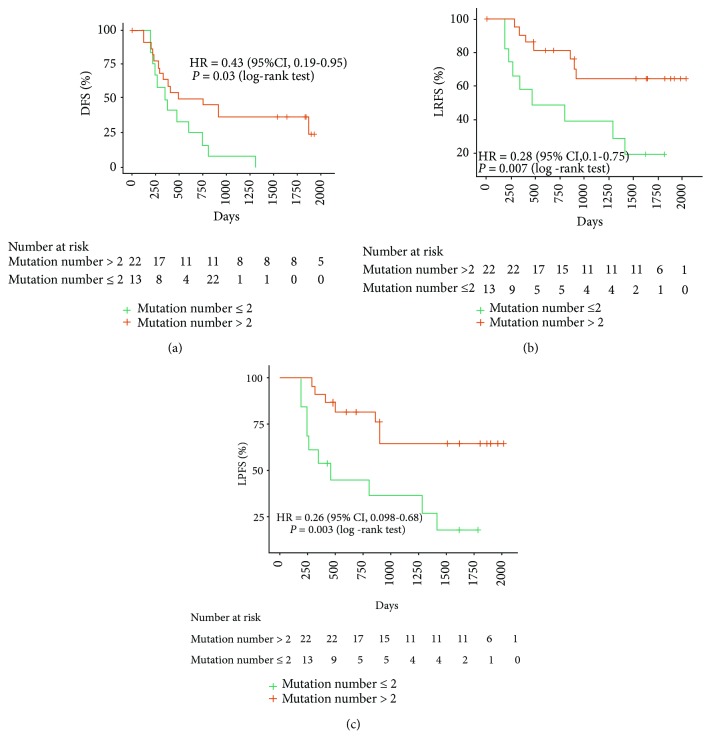
The Kaplan–Meier survival curve estimates the relationship between prognosis and mutation number in stage IIIA-N2 NSCLC patients: (a) disease-free survival (DFS); (b) local-regional recurrence-free survival (LRFS); (c) local-regional progression-free survival (LPFS).

Cox proportional hazards analysis was used to determine the relative contribution of various factors to outcome and showed that smoking, mutation number, and mutation frequency could affect the progression-free survival (PFS). The results revealed that patients with mutation frequency > 0.025 and mutation number > 2 had significantly better PFS (Figures 1(a) and 1(b)). The patients with smoking history had better PFS (Table 1 and Figure 1(c)). Also, Kaplan–Meier curves showed that the overall survival (OS) of the well-differentiated NSCLC patients was better than that of the poorly differentiated NSCLC patients (Figure 1(d)). The median follow-up was 61 months (range, 58 to 64 months). The disease-free survival (DFS), local-regional recurrence-free survival (LRFS), and local-regional progression-free survival (LPFS) in different mutation number groups were listed. There were no significant differences in the rates of OS; however, the rates of DFS, LRFS, and LPFS have significant differences in the mutation number > 2 and mutation number ≤ 2 groups, respectively (Figures 2(a)–2(c)).

### 3.2. Whole-Exome Analysis of Stage IIIA-N2 NSCLC Patients

**Figure 3:**
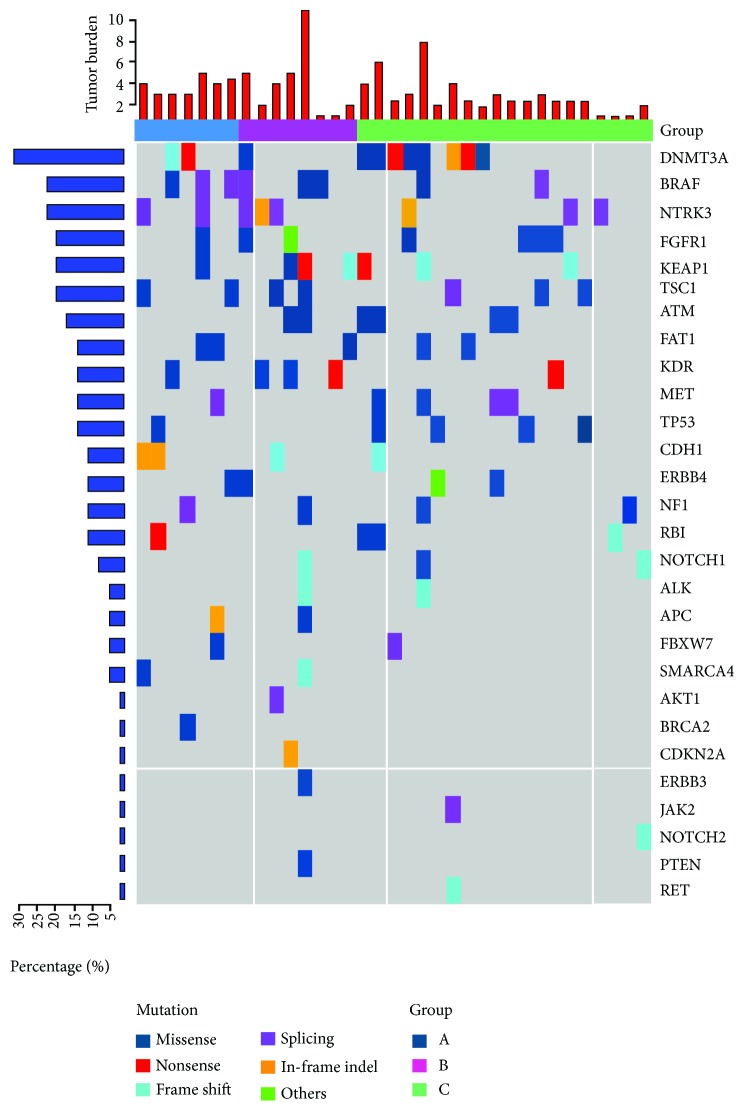
Top list of somatic mutation targets in stage IIIA-N2 NSCLC patients: (a) preoperation; (b) postoperation; (c) postradiochemotherapy.

**Figure 4:**
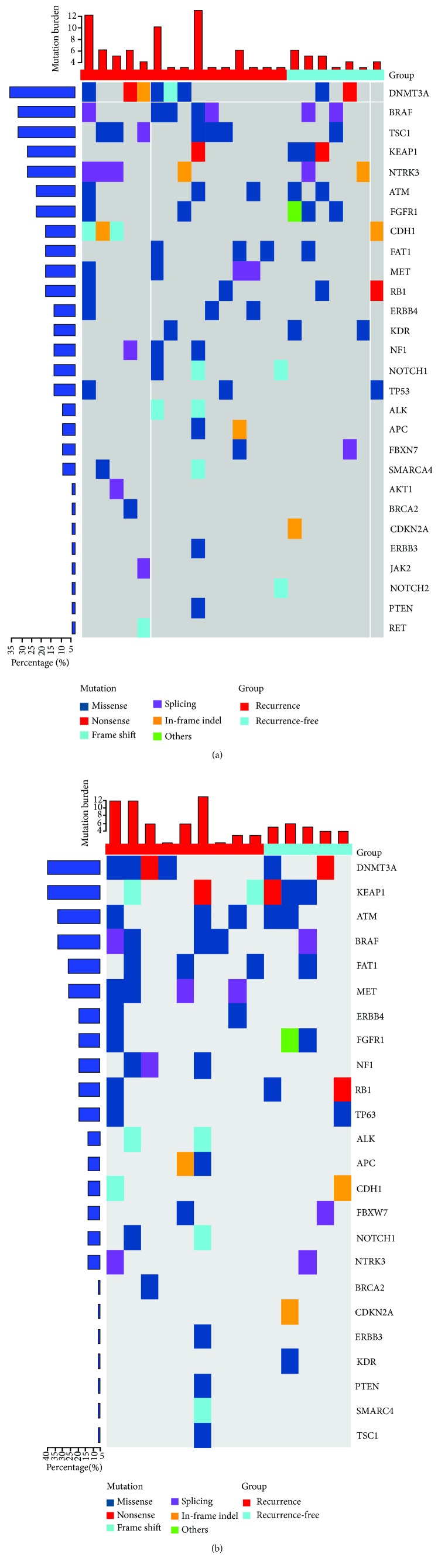
The somatic mutation targets in recurrence and recurrence-free stage IIIA-N2 NSCLC patients: (a) mutation number > 2; (b) mutation frequency > 0.025.

Whole-exome capture and sequencing were performed on plasma from 35 stage IIIA-N2 NSCLC patients. Of the 35 sequenced samples, 7 were derived from the preoperation group and 8 from the postoperation group whereas the remaining 20 samples were from the postradiochemotherapy group (Figure 3). Then, we summarized the somatic mutation targets of mutation number >2 and mutation frequency > 0.025 separately for patients with or without recurrence (Figure 4). We found that compared to recurrence patients, Met, ALK, APC, PTEN, ERBB4, NF1, and other genes in recurrence-free patients never mutated, involving multiple tumor suppressor genes and lung cancer-driven genes.

### 3.3. The Relationship between Molecular Markers and Clinical Features

**Figure 5:**
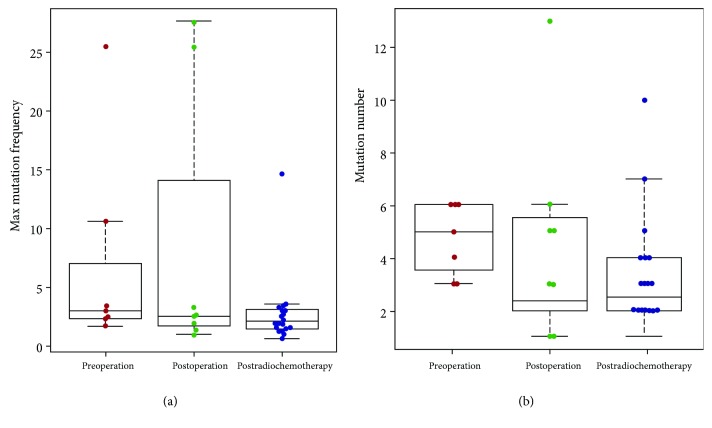
The relationship between molecular indexes and different sample collection times.

**Figure 6:**
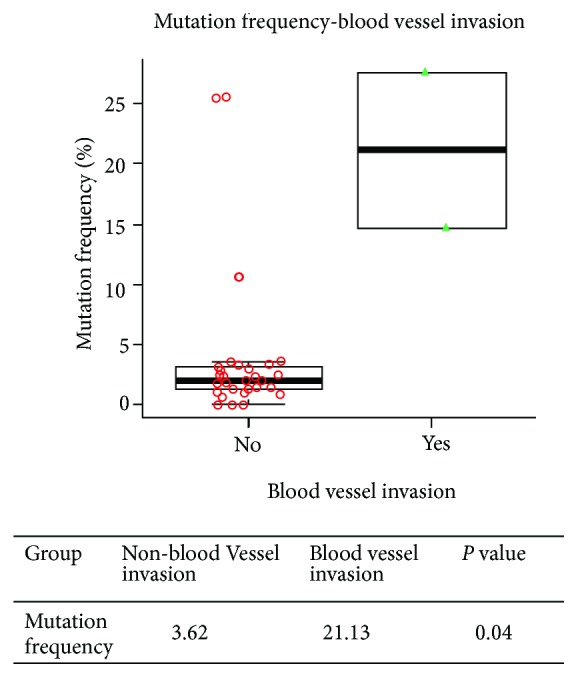
The relevant research on the mean of mutation frequency and blood vessel invasion.

Using a nonparametric comparison method, the differences between the molecular indexes (the mutation number and the highest mutation frequency) of each group were compared using various factors. The analysis showed that the sample collection time had a significant effect on the mutation number and the highest mutation frequency. The mutation number of the preoperation group was higher than that of the postradiochemotherapy group (*P* = 0.023) (Table 2 and Figure 5). The highest mutation frequency was significantly related to the occurrence of blood vessel invasion. The mutation frequency was positively correlated with vascular invasion (*P* = 0.04) (Figure 6).

## 4. Discussion

Extensive clinical studies are currently underway to identify prognostic factors that may affect survival or local recurrence in order to further subdivide N2 patients into subgroups with good or poor prognosis and to guide the individualized implementation of postoperative management. The predictive value of the clinical or pathological factors reported in the literature that affect the survival and local recurrence after complete surgical resection of stage III-N2 NSCLC remains controversial and varies from study to study [10].

In addition to the commonly used clinical or pathological factors, we first introduced the molecular features—the mutation number and the highest mutation frequency in cfDNA in stage IIIA-N2 NSCLC patients—to assess the prognosis. The molecular classification of tumors is conducive to tumor-targeted therapy and individualized treatment. At present, tumor genotyping relies mainly on biopsy or surgical removal of tissue specimens for detection and then for determination of the disease status and treatment response. In most cases, there is spatial and temporal heterogeneity within the tumor [11]. The puncture of a single part of the tissue is not enough to reflect the heterogeneity within the tumor and between different tumor nodules. The pretreatment tumor tissue analysis cannot fully reflect the differences of tumor cells in different treatment stages. And tissue biopsy diagnosis is invasive, with risks such as bleeding after puncture and tumor cell metastasis. Therefore, the use of peripheral blood cfDNA for real-time dynamic tumor monitoring has more and more become the direction of precision medical treatment. Our study found that the mutation number of cfDNA > 2 and the mutation frequency > 0.025 are related to the prognosis of stage IIIA-N2 NSCLC patients.

The pathological type is controversial for predicting survival in patients with stage IIIA-N2 NSCLC after complete resection. It has been reported that the 5-year OS of pN2 NSCLC patients with postoperative pathological adenocarcinoma is 20%, while the 5-year OS of patients with squamous cell carcinoma is 26% [10]. Inoue and associates noted that the histology type of adenocarcinoma was an independent predictor of poor prognosis for patients with pN2 NSCLC after surgery [12]. In addition, studies have found that the risk of recurrence is high in stage IIIA-N2 NSCLC patients with a large number of N2-positive lymph nodes, multilevel N2 involvement, and large tumor size [13]. There is a higher risk of local recurrence of NSCLC with higher T staging. Pathological T staging is one of the important predictors of survival after resection for patients with stage IIIA-N2 disease. The 5-year OS is about 22% for pathological T3 patients and 33% for T1-T2 patients [14, 15]. However, in our study, no significant correlations were found between these factors and prognosis. The reason for the result of our analysis may be that there were no sufficient data to find meaningful indicators. In this study, we found that the higher mutation number in cfDNA was associated with better PFS. However, the study conducted by Haricharan et al. [16] demonstrated that for ER^+^ breast cancer patients, those with high-mutation load tumors exhibited significantly shorter overall survival (*P* = 0.02). It was mainly attributed to those somatic mutations in DDR pathway genes and in ER-related genes, which were associated with worse overall survival, were significantly more in the high-mutation load group than in the low-mutation load group. Our results revealed that patients in the mutation number > 2 group had significantly better PFS than those in the mutation number ≤ 2 group. DFS, LRFS, and LPFS had remarkably significant differences in the mutation number > 2 and mutation number ≤ 2 groups, respectively. What is more, results of many other studies for patients with NSCLC or other types of malignant tumor also confirmed that higher mutation burden is an indicator for longer PFS in the setting of immunotherapy [7–9, 17, 18]. Altered amino acid that resulted from genetic mutations in tumor could serve as a neoantigen and elicit the antitumor immune response. Consequently, the number of mutations could be a prognostic marker for tumor patients. It has been verified that tumors in smokers generally have more tumor mutations compared with those in nonsmokers [19]. This could also explain why smoking status could be a favorable prognostic factor in our research. Our study also demonstrated that the higher mutation frequency was a positive prognostic factor for patients with stage IIIA-N2 NSCLC. This is contrary to the result of the study conducted by Möhrmann et al. [20] who reported that patients with high exosomal nucleic acid mutation allelic frequency (MAF) had shorter median survival compared with patients with low MAF. However, it was clearly indicated in their study that the tools used to estimate disease burden were not optimal so that their study result should be interpreted with caution.

Furthermore, in order to identify specific somatic mutations which are concerned with prognosis, we analyzed the mutation heat map. We found that when the patients were divided into the recurrence group and the recurrence-free group, the different genes between the two groups were concentrated in the genes such as MET, NF1, ALK, APC, PTEN, and ERBB4, involving multiple tumor suppressor genes and lung cancer-driven genes used as targets for targeted therapy.

In addition, we analyzed the relationship between molecular markers and clinical features. The result revealed that the sample collection time had a significant effect on the mutation number and the highest mutation frequency. The mutation number and the highest mutation frequency were higher in the preoperation group than in the other groups. When patients undergo surgery or chemoradiotherapy, the mutation number and mutation frequency in cfDNA significantly reduced, indicating that the treatment was effective. Meanwhile, the highest mutation frequency was closely related to the occurrence of blood vessel invasion. The highest mutation frequency was higher in the blood vessel invasion group. It might be that as the tumor progressed, the tumor burden gradually increased and the mutation frequency also increased in cfDNA.

In conclusion, our findings suggested that mutation number and mutation frequency in cfDNA could be prognostic factors for patients with stage IIIA-N2 NSCLC. Better PFS was associated with mutation number of cfDNA > 2 and the mutation frequency > 0.025. Mutation number and mutation frequency would be promising to be tools for effectiveness evaluation for surgery and radiochemotherapy. Future studies with large samples are warranted to verify its utility value in the future clinical practice.

## Figures and Tables

**Table 1:** Cox proportional hazards analysis for progression-free survival in stage IIIA-N2 NSCLC patients.

	*β*	Sx	Wald	OR (95% CI)	*P*
Gender (male, female)	0.31966	0.388585	0.822626	1.4 (0.64-2.9)	0.417
Age, yr (≤60, >60)	-0.37059	0.496416	-0.74654	0.69 (0.26-1.8)	0.438
Smoking (Y, N)	-0.83781	0.394112	-2.12581	0.43 (0.2-0.94)	**0.031**
KPS (>80, ≤80)	-0.24517	0.388775	-0.63062	0.78 (0.37-1.7)	0.527
Largest tumor diameter (<3, 3-4.9, and ≥5)	-0.02777	0.28055	-0.099	0.97 (0.56-1.7)	0.921
Histology (adenocarcinoma, nonadenocarcinoma)	0.15502	0.470391	0.329556	1.2 (0.46-2.9)	0.738
pT status (T1-2, T3)	-0.23663	0.543001	-0.43579	0.79 (0.27-2.3)	0.654
cN2 (Y, N)	0.356603	0.380638	0.937093	1.4 (0.68-3)	0.350
Tumor differentiation (well differentiated, poorly differentiated)	-0.57978	0.484415	-1.19686	0.56 (0.22-1.4)	0.253
Met mutation (Y, N)	0.431686	0.507633	0.850389	1.5 (0.57-4.2)	0.415
Mutation number > 2	-0.88498	0.397069	-2.22878	0.41 (0.19-0.9)	**0.028**
Mutation frequency > 0.025	-0.78888	0.407384	-1.93645	0.45 (0.2-1)	**0.045**

**Table 2:** The comparative analysis on the mean of molecular indexes and different sample collection time groups.

Group	Preoperation	Postoperation	Postradiochemotherapy	*P* value (preoperation vs. postoperation)	*P* value (preoperation vs. postradiochemotherapy)	*P* value (postoperation vs. postradiochemotherapy)
Mutation number	5 (4.71)^∗^	4 (4.62)	2.5 (3.15)	0.4775	**0.023**	0.313
Mutation frequency	2.99 (7.01)	2.6 (8.24)	1.85 (2.68)	0.6431	0.285	**0.067**

^∗^Median ($\bar{X}$).

## Data Availability

The original data supporting the findings of this study are currently under embargo because of ethical requirements. Requests for data will be considered by the corresponding authors after the publication of this article.

## Abbreviations

NSCLC: Non-small-cell lung cancer
cfDNA: Cell-free DNA
OS: Overall survival
PFS: Progression-free survival
DFS: Disease-free survival
LRFS: Local-regional recurrence-free survival
LPFS: Local-regional progression-free survival.
